# The Emerging Role of the Brain–Gut Axis in Amyotrophic Lateral Sclerosis: Pathogenesis, Mechanisms, and Therapeutic Perspectives

**DOI:** 10.3390/ijms26178419

**Published:** 2025-08-29

**Authors:** Eun Jin Yang

**Affiliations:** Department of KM Science Research, Korea Institute of Oriental Medicine (KIOM), Yuseong-daero 1672, Yuseong-gu, Daejeon 34054, Republic of Korea; yej4823@gmail.com

**Keywords:** amyotrophic lateral sclerosis, brain–gut axis, central nervous system, therapy

## Abstract

Amyotrophic lateral sclerosis (ALS) is a fatal neurodegenerative disease characterized by progressive loss of motor neurons. Although genetic and environmental factors are established contributors, recent research has highlighted the critical role of the gut–brain axis (GBA) in ALS pathogenesis. The GBA is a bidirectional communication network involving neural, immune, and endocrine pathways that connect the gut microbiota with the central nervous system. Dysbiosis in ALS disrupts this axis, leading to increased intestinal permeability, neuroinflammation, and excitotoxicity. Notably, reductions in butyrate-producing bacteria, alterations in microbial metabolites, and enhanced NLRP3 inflammasome activation have been observed in patients with ALS. These changes may precede motor symptoms, suggesting a potential causative role. Interventions targeting the microbiome, such as dietary modulation, have shown promise in delaying disease onset and reducing inflammation. However, the clinical evidence remains limited. Given that gut dysbiosis may precede neurological symptoms, microbiota-targeted therapies offer a novel and potentially modifiable approach to ALS treatment. Understanding the role of GBA in ALS will open new avenues for early diagnosis and intervention. Further clinical trials are required to clarify the causal links and evaluate the efficacy of microbiome-based interventions. Understanding the brain–gut–microbiota axis in ALS could lead to new diagnostic biomarkers and therapeutic strategies.

## 1. Introduction

Amyotrophic lateral sclerosis (ALS) is a progressive and fatal neurodegenerative disease that primarily targets motor neurons, which are critical for conveying signals from the brain and spinal cord to the skeletal muscles, leading to progressive muscle weakness, atrophy, and eventual paralysis [1]. In recent decades, extensive research has demonstrated that the cause of ALS is based on genetic and environmental factors, which play a role in triggering and advancing the disease. Several studies have explored the characteristics and pathological mechanisms associated with ALS, including neuroinflammation, RNA metabolism, mitochondrial dysfunction, and altered synaptic function [2]. In ALS, the primary pathological changes are associated with the motor pathways of the central nervous system (CNS). The loss of motor neurons is a hallmark of ALS, as it reduces the functional connection between the brain and muscles.

Neuroinflammation is a pathological mechanism that leads to motor neuron death and is involved in glial activation. Microglia and astrocytes are glial cells that act as immune cells and are activated during ALS. Activated glial cells release inflammatory cytokines, reactive oxygen species (ROS), and other neurotoxic substances, and induce motor neuron death, thereby accelerating the progression of ALS [3]. Furthermore, reactive astrocytes and microglia are involved in increasing glutamate levels, leading to glutamate excitotoxicity in neurons [4]. In a previous study, activation of astrocytes led to an increase in NLRP3 inflammasome expression, which elevated levels of IL-1β and TGF-β1. This, in turn, induced neuroinflammation and contributed to neuronal death in hSOD1^G93A^ mice [5]. Furthermore, elevated levels of inflammatory markers have been observed even during the pre-symptomatic stages of ALS, suggesting that inflammation may contribute to the initiation of the disease rather than being solely a secondary consequence of neurodegeneration [6]; early immune activation implies that neuroinflammatory processes are likely involved at the onset of ALS pathogenesis, highlighting a potential therapeutic window for early intervention before significant motor neuron loss occurs [7].

In recent years, there has been growing scientific interest in the gut microbiota and its influence on various aspects of human health, including neurodegenerative diseases [8]. The gut–brain axis (GBA) represents a dynamic, bidirectional communication network between the gastrointestinal (GI) tract and the CNS, involving neural, immune, endocrine, and metabolic signaling pathways. Among the various modulators of this axis, the gut microbiota has emerged as a particularly influential and modifiable factor capable of shaping systemic and neurological outcomes. Gut microbes influence brain function by acting on neuronal and glial cells and producing a wide array of bioactive molecules, including metabolites, neurotransmitters, and immune mediators. Notably, short-chain fatty acids (SCFAs), generated via microbial fermentation of dietary fiber, have been recognized for their neuroprotective and anti-inflammatory properties. Diverse and well-balanced gut microbiota, often supported by a plant-based, fiber-rich diet, is essential for optimal SCFA production [9]. Emerging evidence indicates that SCFAs may modulate brain function through the GBA, suggesting a potential role in the prevention or mitigation of neurodegeneration. Therapeutic strategies aimed at modulating the microbiota-GBA hold significant promise for the management of neurodegenerative diseases, marking a novel frontier in the era of precision and systems medicine.

This review aims to provide a comprehensive overview of the GBA mechanisms involved in ALS and highlight the therapeutic potential of microbiota-based interventions.

## 2. The Impact of the Gut Microbiome on the Nervous System

The gut microbiome, including its microorganisms, is important for maintaining physiological homeostasis in health. In addition, several reports have demonstrated that the gut microbiome interacts bidirectionally with the CNS via the GBA in mental health disorders such as depression, autism spectrum disorders, and neurodegenerative diseases, including Alzheimer’s and Parkinson’s disease [10]. The GBA is induced by the vagus nerve, immune responses, and metabolite signaling. Several studies have shown that the GBA and dysbiosis of the gut microbiota play a significant role in neurodegenerative diseases [11,12,13].

**Figure 1:**
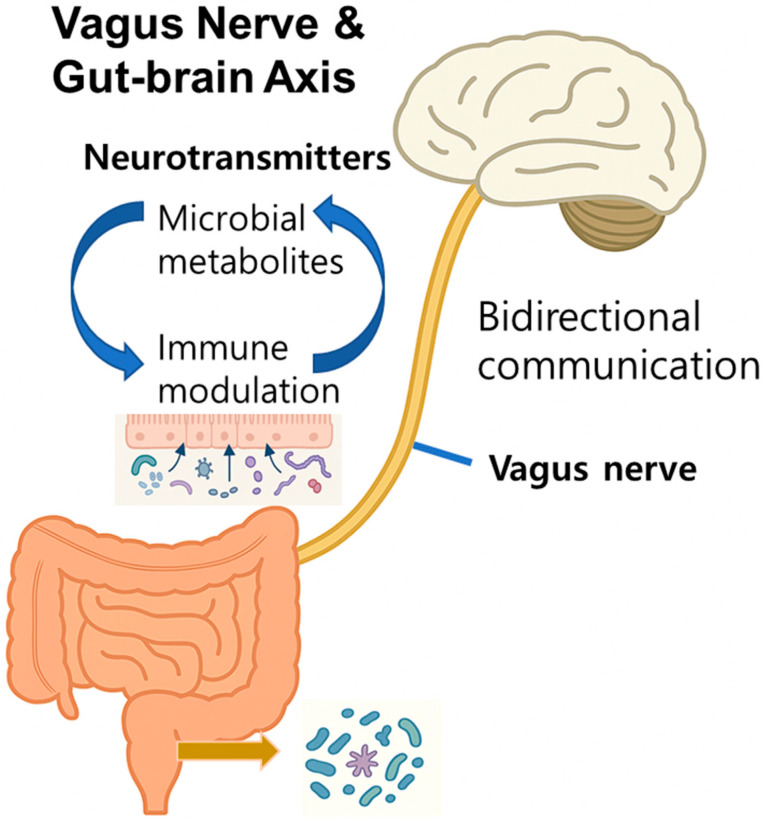
Gut microbiota and gut–brain axis in health and disease. Summary of the bidirectional communication between the gut microbiota and brain via neural, immune, and endocrine pathways. Microbial metabolites, neurotransmitters, and immune signals modulate central nervous system (CNS) function, whereas factors such as diet, stress, and medication influence microbiota composition. Disruptions in these pathway axes are linked to neurodegenerative and metabolic disorders.

The bidirectional communication between the GI microbiota and the CNS is orchestrated through the integrated functions of the nervous, immune, and endocrine systems (Figure 1). This axis plays a crucial role in maintaining homeostasis within the gut, brain, and microbial ecosystems, thereby contributing significantly to physical and mental health. The regulatory processes of the GBA are mediated by various signaling molecules, including microbial-derived metabolites, neurotransmitters, and hormones. Although the full spectrum of its mechanisms remains to be elucidated, current evidence highlights several core components of the GBA that offer promising avenues for therapeutic intervention, particularly in the context of neurodevelopmental disorders [9,14,15].

The gut microbiota encompasses a dynamic and complex community of microorganisms residing within the GI tract of animals, including humans. They are predominantly composed of bacteria, but also include fungi, archaea, parasites, and viruses, albeit in smaller proportions [16]. Over millennia, these microorganisms have co-evolved with their hosts, forming intricate symbiotic relationships that are essential for host physiology [17]. The aggregate genetic content of these microbes, termed the gut microbiome, comprises approximately 3.3 million genes, surpassing the human genome by an order of magnitude [17,18]. Moreover, the estimated number of microbial cells in the human gut is roughly equivalent to that of human somatic cells, significantly enhancing the metabolic capabilities analogous to those of the liver [19]. The human gut microbiota is predominantly composed of four bacterial phyla, Firmicutes, Bacteroidetes, Proteobacteria, and Actinobacteria. Firmicutes and Bacteroidetes account for approximately 90% of the total microbial population. Minor phyla, including *Fusobacteria* and *Verrucomicrobia*, are also present [20,21]. Microbial density and composition vary throughout the GI tract and are influenced by chemical, nutritional, and immunological factors. For instance, the highly acidic environment of the stomach, as well as the elevated pH and rapid transit time of the small intestine, limit microbial colonization [22]. In contrast, the colon provides an optimal anaerobic environment, slower transit, and substrates for fermentation, supporting a high density and diversity of microbiota [22]. This regional heterogeneity highlights the importance of anatomical context in microbiota analysis.

Intra-individual gut microbiota are not static; they are modifiable and respond dynamically to intrinsic factors, such as host genetics, age, and health status, as well as extrinsic factors, including diet, pharmacological agents, lifestyle, physical activity, infections, stress, and geographic location [19]. Inter-individual variability has also been noted, with each healthy individual harboring a unique microbial community. The concept of a “core microbiota” refers to a set of microbial taxa consistently found across individuals. However, the greater conservation of microbial gene functions than taxonomic profiles suggests that defining the core microbiota at the functional level may be more appropriate [23].

The gut microbiota exerts a profound influence on host immune regulation, digestive processes, metabolic pathways, and even neurological signaling [24]. Among various metrics, the Firmicutes/Bacteroidetes (F/B) ratio has been proposed as a potential indicator of gut microbial health, useful in disease risk assessment and therapeutic targeting [25]. Notably, an elevated F/B ratio has been associated with metabolic and neurodegenerative disorders such as type 2 diabetes (T2D), obesity, and dementia [25,26,27].

## 3. The Importance of the GBA

### 3.1. Neural Pathways: The Vagus Nerve (A Key Mediator of the GBA)

The vagus nerve, the longest cranial nerve, is the primary communication channel between the gut and brain [28]. It is a major component of the peripheral nervous system (PNS) and is composed of approximately 80% afferent and 20% efferent fibers. In addition, it mediates bidirectional communication between visceral organs—such as the GI, cardiovascular, and respiratory systems—and the CNS, playing key roles in regulating appetite, stress, inflammation, and cognition [29]. Gut-derived signals, including neurotransmitters such as serotonin (5-HT) and peptides, activate the vagus nerve and relay information to the brain, influencing mood, stress responses, and emotional regulation. Disruption of the vagus nerve impairs the ability of the gut microbiome to modulate brain activity [28]. Thus, this pathway is crucial for understanding how changes in the gut microbiota can alter behavior and cognition.

Previous experimental studies have indicated that the vagus nerve integrity is essential for hippocampal neurogenesis and stress resilience. Vagal disruption impairs cognition and activates microglia, whereas vagus nerve stimulation enhances brain-derived neurotrophic factor expression, synaptic plasticity, and memory performance [30,31,32].

### 3.2. Immune Pathways: Immunomodulatory Roles of SCFAs in the GBA

**Figure 2:**
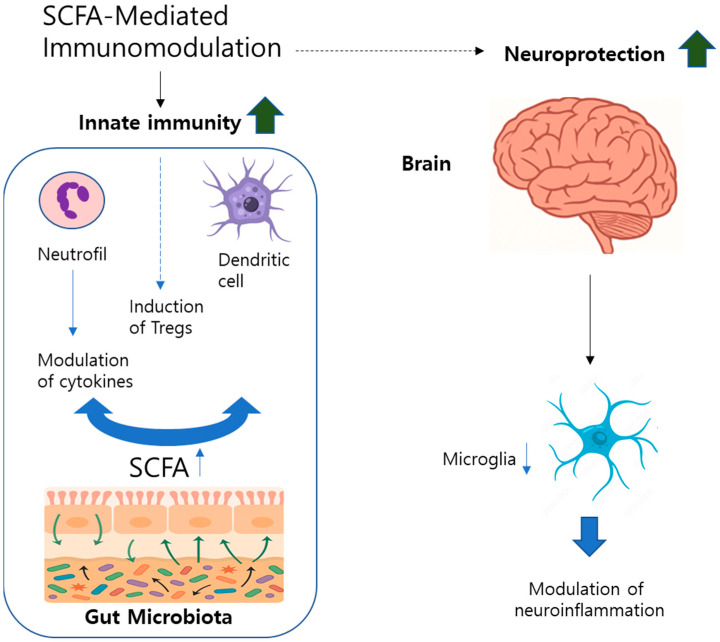
Short-chain fatty acid (SCFA)-mediated immune and neuroinflammatory modulation in the gut–brain axis. Gut microbiota produce SCFAs, which influence the innate immune system, modulating neutrophil and dendritic cell activity, promoting Treg induction, and strengthening intestinal barrier integrity. SCFAs also act on microglia in the CNS, reducing neuroinflammation and contributing to neuroprotection.

The immune and inflammatory responses significantly contribute to the pathogenesis of neurodegenerative disorders through interactions among inflammatory mediators, immune cells, and neuronal pathways [33]. Among SCFAs, butyrate—produced by the fermentation of dietary fibers—plays a particularly important role in maintaining intestinal barrier integrity and modulating neuroinflammation [34,35] (Figure 2). SCFAs influence innate immunity, primarily by regulating neutrophil functions, including cytokine secretion, chemotaxis, and ROS production. These actions occur via the inhibition of histone deacetylases (HDACs) and binding to specific receptors such as free fatty acid receptor 2 (FFAR2) [36]. In adaptive immunity, SCFAs prevent monocyte differentiation into macrophages and dendritic cells, impair antigen uptake, and suppress inflammatory cytokine release [37]. In particular, butyrate promotes regulatory T cell (Treg) differentiation through activation of GPR109A receptors on dendritic cells, enhancing immune tolerance [38]. However, direct HDAC inhibition by SCFAs may also facilitate differentiation toward pro-inflammatory Th1 and Th17 cell phenotypes via mTOR signaling [39]. Experimental studies have demonstrated that SCFA administration reduces neutrophil infiltration and inflammation in colitis models; however, the precise mechanisms remain unclear [37]. In human clinical studies, prebiotic and synbiotic supplementation has been shown to lower systemic inflammatory biomarkers, including C-reactive protein and TNF-α, supporting potential therapeutic implications [40]. Additionally, SCFAs strengthen the intestinal epithelial defense by promoting antimicrobial peptide secretion and modulating cytokines such as IL-18 [41]. Butyrate specifically exhibits potent anti-inflammatory effects in intestinal macrophages by inhibiting nitric oxide, IL-6, and IL-12 production through HDAC-dependent pathways, suggesting therapeutic potential for inflammatory bowel disease (IBD) [37]. Reduced levels of butyrate-producing bacteria and transporters have been observed in IBD and are correlated with increased mucosal inflammation [38,42].

Within the CNS, microbiota-derived SCFAs modulate microglial maturation and function, directly influencing neuroinflammatory pathways linked to neurodegeneration [43,44]. Disruptions of microbiota composition via antibiotic treatment lead to abnormal microglial activation and heightened neuroinflammation, whereas SCFA supplementation promotes anti-inflammatory and neuroprotective microglial responses [45,46]. These findings highlight the critical role of SCFAs in gut–brain–immune axis regulation, suggesting therapeutic opportunities for managing inflammation-related neurodegenerative diseases.

### 3.3. Enteroendocrine Signaling in GBA Regulation

Two specialized neuroendocrine cell populations in the intestinal epithelium regulate gut–brain communication by secreting signaling peptides into nearby blood vessels and afferent nerve fibers. Enteroendocrine cells (EECs), which are widely distributed throughout the GI tract with a density that increases distally, represent the largest endocrine organs in the body [47,48]. EECs express various chemosensory receptors, such as G-protein-coupled receptors (GPCRs) and nutrient transporters, enabling the detection of luminal nutrients. Upon activation, EECs secrete peptides including cholecystokinin (CCK), peptide YY (PYY), glucagon-like peptide-1 (GLP-1), and glucose-dependent insulinotropic polypeptide (GIP), which collectively regulate insulin secretion and energy homeostasis [47,48,49]. Enterochromaffin cells (ECs) produce approximately 95% of the body’s 5-HT, which influences local gut neurons that express 5-HT receptors [49,50]. Additionally, gastric X/A-like cells (P/D1 cells) secrete ghrelin, which acts via the ghrelin receptor (GHSR1a) to modulate appetite and energy balance [51,52].

Gut peptides communicate with the CNS via two primary mechanisms: paracrine signaling through vagal afferents projecting to the nucleus tractus solitarius (NTS) and endocrine signaling through systemic circulation directly to the brainstem [52,53,54].

SCFAs, produced by gut microbiota, regulate EEC activity by binding to GPCRs (e.g., GPR41 and GPR43), stimulating the secretion of GLP-1, PYY, gamma-aminobutyric acid (GABA), and 5-HT [55,56,57]. Specifically, butyrate enhances 5-HT production by upregulating tryptophan hydroxylase 1 in ECs [58,59]. Germ-free animal studies have demonstrated significantly reduced 5-HT levels, which are reversible by microbiota colonization, highlighting the essential role of microbiota in regulating gut-derived 5-HT synthesis [60].

## 4. The Brain-Gut-Microbiome Axis in ALS

**Figure 3:**
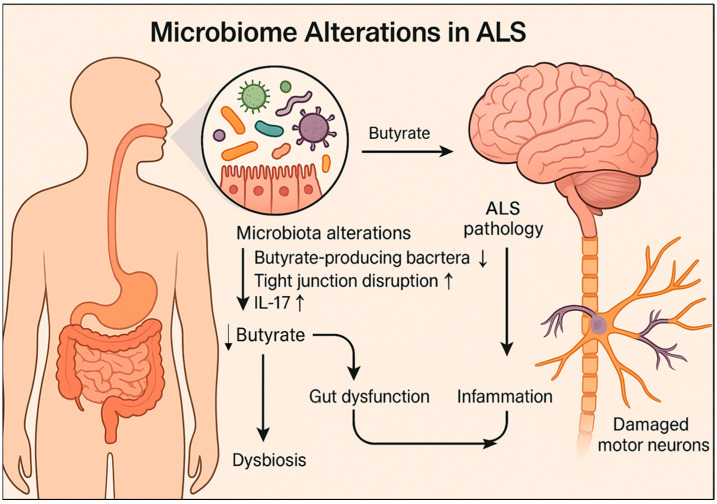
The proposed mechanisms by which gut microbiota alterations contribute to amyotrophic lateral sclerosis progression. Early dysbiosis, marked by a reduction in butyrate-producing bacteria, such as *Butyrivibrio fibrisolvens*, leads to impaired intestinal barrier integrity and increased pro-inflammatory cytokine IL-17 levels.

Emerging evidence suggests that changes in the gut microbiome may precede neuromuscular symptoms in ALS and influence its progression [58,59]. In a previous study using a SOD1^G93A^ mouse model, notable reductions in butyrate-producing bacteria, such as *Butyrivibrio fibrisolvens*, were identified, along with disrupted intestinal tight junctions and increased pro-inflammatory cytokine IL-17 levels [61,62] (Figure 3). These intestinal abnormalities appeared in young mice before the onset of ALS symptoms, suggesting early mucosal barrier dysfunction [62]. Additionally, significant abnormalities in the enteric nervous system (ENS)—which is crucial for gut motility and microbiome interaction [63]—were observed in G93A mice [62].

Butyrate supplementation delayed disease onset, improved ENS function and muscle strength, and extended survival in G93A mice [62]. These results indicate that microbiome modulation is a promising therapeutic strategy for ALS. Similar microbiome-dependent variability affecting inflammation and ALS phenotypes has been observed in C9orf72-null mice across different facilities [64]. Antibiotic treatment and fecal microbiota transplantation (FMT) mitigated inflammatory markers, autoimmune responses, and spinal cord immune cell infiltration [64]. Additionally, in a mouse model expressing human mutant TDP43, intestinal dysfunction and altered glial fibrillary acidic protein (GFAP) and α-smooth muscle actin (α-SMA) expressions preceded neuromuscular symptoms [65]; butyrate treatment significantly delayed disease onset in TDP43 mice [65]. Human studies also support intestinal dysbiosis and systemic microbial infections as early indicators of ALS [66,67,68]. For example, Blacher et al. [69] reported a significant reduction in *Prevotella* spp. and an increase in pro-inflammatory microbes in patients with ALS. Other clinical studies have identified altered gut microbial profiles and elevated systemic inflammatory markers in ALS cohorts [67,68], reinforcing the hypothesis that gut dysbiosis may contribute to disease onset and progression. Collectively, these findings highlight impaired gut function and inflammation as potential contributors to the pathogenesis of ALS, underscoring the importance of microbiome modulation as a therapeutic target.

Interest in the gut virome and mycobiome is growing, although their roles in ALS remain unclear. Chronic fungal exposure and fungal toxins (mycotoxins) have been shown to induce ALS-like symptoms in animal models, although direct evidence in humans is limited [70]. Moreover, reduced diversity and compositional shifts in archaeal communities, such as elevated levels of *Methanobrevibacter*, have been observed in patients with ALS, warranting further investigation [71].

### 4.1. Neural Pathway of the Brain–Gut–Microbiota Axis

Recent studies have suggested that ECs and the vagus nerve may serve as key mediators in the transmission of microbial signals from the gut to the CNS in patients with ALS. ECs function as bidirectional sensors that detect microbial or dietary stimuli on their luminal side and release neuroactive substances such as 5-HT and histamine, which activate vagal afferent terminals in the lamina propria [72,73].

In a murine model, localized intestinal infection with *Campylobacter jejuni* activated vagal sensory neurons and subsequently stimulated the NTS, a primary visceral sensory relay center in the brain [74]. Furthermore, vagal nerve injury has been shown to reduce hippocampal microglial activation, suggesting that vagal signaling influences brain immune tone [75]. These findings support the hypothesis that early microbial changes in ALS can trigger vagus-mediated immune signaling into the brain, potentially contributing to chronic neuroinflammation.

**Figure 4:**
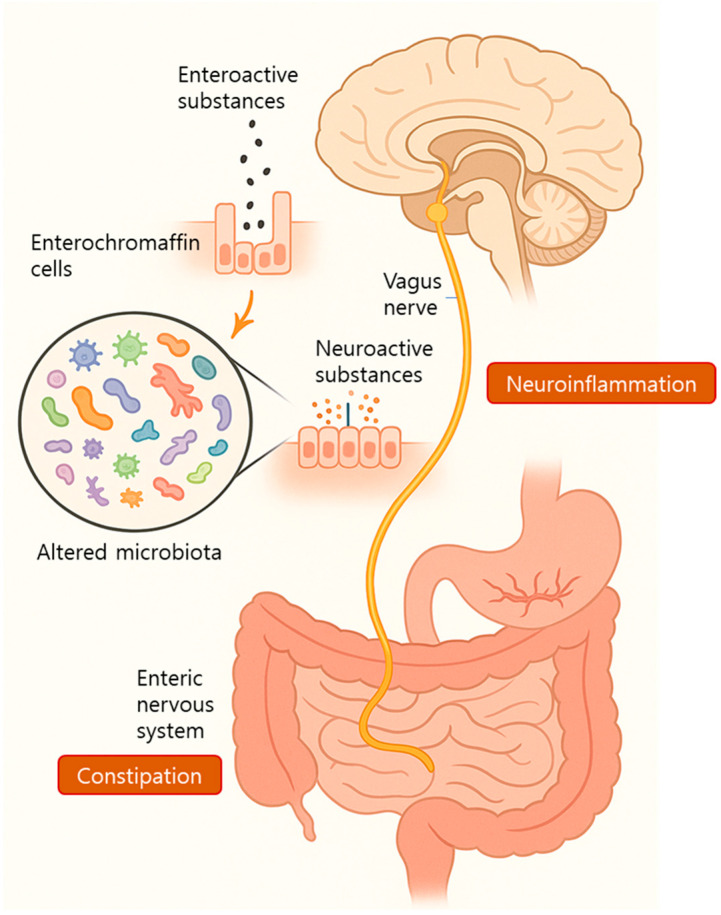
An illustration depicting the neural pathway of the brain–gut–microbiota axis in patients with amyotrophic lateral sclerosis (ALS). Altered gut microbiota interacts with enterochromaffin cells, leading to the release of neuroactive substances such as serotonin that signal through the vagus nerve to the brain. These signals contribute to neuroinflammation. Additionally, changes in microbial metabolites influence ENS activity and may contribute to constipation, a common symptom in patients with ALS.

Constipation, a frequent symptom in patients with ALS, is often attributed to decreased intestinal motility. This motility is primarily regulated by the autonomic nervous system (ANS) and ENS [76]. Intrinsic sensory neurons within the GI tract, such as Dogiel type I and II neurons, serve as targets for microbial metabolites, including, SCFAs, chemotactic peptides, and tryptamine, all of which influence intestinal transit via ENS modulation [72,77] (Figure 4). Therefore, it is plausible that altered microbial metabolite profiles in ALS contribute to impaired ENS function and subsequent constipation. Notably, the ENS shares many structural and neurochemical features with the CNS [63]. Remarkably, Kulkarni et al. [78] identified ongoing neurogenesis and neuronal remodeling in the adult ENS, suggesting a capacity for dynamic adaptation. Because the gut microbiome is itself in constant flux, it may directly modulate ENS neuronal populations during ALS progression, potentially altering gut function and symptomatology.

ALS progression itself can reciprocally influence the composition of the gut microbiota. As ALS advances, patients often experience impaired chewing, swallowing, and intestinal motility, which, in turn, alters the gut environment. These physiological changes, along with dietary modifications such as reduced intake or gastrostomy resulting from dysphagia, may affect the quantity, quality, and variety of nutrients reaching the intestine, thereby influencing microbial diversity and abundance [79]. Additionally, slowed intestinal transit may permit the overgrowth of certain microbial species, disrupting the microbial equilibrium [80]. Dysfunctional secretion of mucus, bicarbonate, and water, which are key components of the intestinal mucosal environment, can further compromise microbial habitats and alter microbial composition. Furthermore, Macfarlane et al. [81] proposed that ANS dysfunction in ALS may directly affect microbial populations by altering gut physiology. Taken together, these findings emphasize the need to consider bacteria, viruses, fungi, and archaea in ALS-related microbiome research. Investigating these host–microbe interactions and their impact on immune dysregulation and neuroinflammation may illuminate new mechanisms of ALS pathogenesis and therapeutic targets.

### 4.2. Immune Pathway of the Brain–Gut–Microbiota Axis

ALS progression and patient survival may be significantly influenced by gut microbiota-induced immune and inflammatory responses. In C9orf72 mouse models, broad-spectrum antibiotic treatment has been shown to reduce inflammation and autoimmune phenotypes, pre- and post-symptom onset. These effects were later attributed to alterations in the gut microbial composition [64]. Gut-derived microbial signals can promote peripheral and central inflammation, potentially affecting neuronal survival. Notably, lipopolysaccharides (LPS), a major microbial byproduct, have been implicated in peripheral inflammation in ALS. Elevated plasma LPS levels have been reported in patients with ALS compared to healthy controls, despite the absence of active infections, suggesting a GI origin [82]. This is further supported by evidence of increased intestinal permeability in SOD1^G93A^ mice, indicating that a compromised mucosal barrier is a possible source of systemic LPS [62].

The NLRP3 inflammasome, a crucial component of the innate immunity, has been increasingly implicated in the pathogenesis of ALS. Elevated levels of NLRP3 and IL-1β have been detected in pre-symptomatic SOD1^G93A^ mice, with stronger expression observed at 14 weeks of age [83]. In addition, enhanced NLRP3 expression was identified in the dorsal thalamic nucleus and neurons of these mice, suggesting a potential role in subcortical neurodegeneration linked to cognitive dysfunction [84]. Moreover, reducing 17β-estradiol, a hormone upregulated by inflammasome activation, decreased motor neuron loss in SOD1^G93A^ mice [85]. Furthermore, elevated levels of NLRP3, ASC, IL-18, and caspase-1 have been confirmed in patients with ALS, highlighting the translational relevance of these findings [86,87]. Activation of the NLRP3 inflammasome involves two stages, priming and activation, triggered by signals such as oxidative stress, lysosomal damage, or calcium influx [88,89]. Caspase-1 activation leads to pore formation in the cell membrane, promoting the release of inflammatory cytokines and triggering apoptosis. Gut microbiota metabolites play opposing roles in this process: trimethylamine-N-oxide (TMAO) promotes NLRP3 activation through TLR4 signaling, whereas SCFAs inhibit overexpression of inflammasome components such as ASC, NLRP3, IL-1β, and caspase-1 [90,91]. Therefore, persistent NLRP3 overactivation may drive chronic neuroinflammation and exacerbate ALS progression [92]. Thus, targeting the NLRP3 pathway and understanding these intersecting pathways may uncover new therapeutic targets and strategies.

The bidirectional communication between the gut microbiota and CNS is mediated in part by immune pathways. The intestinal mucosal immune system, comprising epithelial cells, immune cells, lymphoid tissue, and resident microbiota, is essential for maintaining local immune balance. Paneth cells, located at the base of intestinal crypts, play a pivotal role in microbial regulation by secreting antimicrobial peptides, such as defensins. Dysfunction of Paneth cells, as evidenced by decreased defensin 5α and increased numbers of abnormal Paneth cells in SOD1^G93A^ mice, may impair microbial homeostasis [62].

Moreover, activated inflammasomes appear to influence the gut microbial composition. For instance, caspase-1 knockout mice display a reduced *Sclerotinia*-to-*Bacteroides* ratio, suggesting that NLRP3 activation may promote microbial imbalance [93]. Clinical data also support these findings; patients with ALS exhibit elevated fecal levels of immune markers such as secretory IgA (sIgA), calmodulin, and eosinophils, indicating that adaptive immune activation may further shape gut microbial communities [66]. Thus, understanding these interactions may also provide new therapeutic targets.

### 4.3. Endocrine Pathway of the Brain-Gut-Microbiota Axis in ALS

In ALS models, reduced butyrate-producing bacteria have been correlated with increased intestinal permeability, whereas butyrate treatment has been shown to enhance tight junction protein expression and delay disease onset [94]. Butyrate has also been shown to stimulate MUC-2 expression, contributing to mucus layer integrity and reduced inflammation [95]. Furthermore, it upregulates claudin-1 and synaptopodin, key components in epithelial barrier maintenance [96]. These findings support the therapeutic potential of SCFAs, particularly butyrate, in restoring intestinal homeostasis and modulating ALS progression.

SCFAs further regulate inflammation by acting on Tregs and microglia. Patients with rapidly progressing ALS show reduced Treg counts and lower FOXP3 expression, a key Treg transcription factor [97]. Butyrate promotes Treg differentiation by inhibiting HDAC and activating GPR109A signaling [98,99]. In microglia, SCFAs suppress pro-inflammatory cytokines (IL-6, IL-1β, and TNF-α) and increase anti-inflammatory cytokines (TGF-β and IL-4), reducing neuroinflammation [71]. Butyrate also downregulates IBA1 expression and IL-17 and LPS levels in SOD1^G93A^ mice, further supporting its role in dampening neuroinflammatory pathways [100]. Moreover, SCFAs influence the ENS and ANS. They act through GPR41/43 receptors expressed in the myenteric plexus and ganglia and modulate vagal nerve activity and intestinal motility [101]. Butyrate enhances ENS function by increasing cholinergic neurons and promoting colonic motility [102]. Overall, SCFAs exert multifaceted effects on immune regulation, intestinal integrity, and neuroinflammation in patients with ALS. Although preclinical studies are promising, further clinical research is required to validate their efficacy in modifying core ALS symptoms [103].

The brain–gut–microbiota axis, an intricate communication network involving the neural, immune, and endocrine systems, has emerged as a promising framework for understanding the pathogenesis of ALS. Dysbiosis of the gut microbiota has been consistently observed in patients with ALS and in animal models; however, the precise mechanisms remain unclear. Further research is necessary to elucidate how gut microbial changes contribute to ALS progression and to bridge the gap between animal model findings and human clinical outcomes. Advances in this field may enhance diagnostic and therapeutic strategies. Although interventions such as FMT, prebiotics, and probiotics remain in their early research stages, they represent potential approaches for restoring microbial balance and improving ALS outcomes [104]. Initial microbiome research with regard to ALS has consistently reported dysbiosis characterized by the overgrowth of potentially pathogenic bacteria and a reduction in microbial diversity [105]. This imbalance may disrupt the intestinal epithelial barrier and trigger immune and inflammatory responses, thereby contributing to the pathogenesis of ALS. Interestingly, increased microbial richness and a higher F/B ratio have been associated with shorter survival in patients with ALS at later stages of the disease [106], although conflicting findings exist, with some studies reporting no significant changes in gut microbiota composition [107].

Given the inflammatory components of ALS, several studies have investigated how gut microbial alterations affect the disease phenotype. Even in the context of genetic susceptibility, such as C9ORF72 mutations, microbiome modulation was found to attenuate inflammatory responses [64]. A longitudinal study observed distinct shifts in gut microbiota composition during ALS progression, showing a decline in beneficial taxa and an increase in potentially neurotoxic bacteria [108]. A reduction in butyrate-producing bacteria has been consistently reported in patients with ALS [109,110,111], although the magnitude and consistency of this reduction varies between studies depending on the patient cohort, regional microbiota diversity, and sampling methodologies [112,113]. These inconsistencies highlight the need for standardized gut microbiome profiling protocols in ALS research. Moreover, although animal models such as SOD1^G93A^ mice have demonstrated clear microbial and inflammatory changes, translation to human ALS cases remains limited. This gap between preclinical and clinical observations is rarely addressed in existing reviews, and this review aimed to highlight both convergence and divergence across model systems.

Furthermore, some previous reports have also found no significant differences in microbial composition between patients with ALS and the relative controls [107], suggesting that dysbiosis may not be universally present and could reflect secondary changes due to disease progression, nutrition, or medication use rather than causal factors.

Microbial imbalance in ALS has also been correlated with elevated inflammatory markers, such as calprotectin, sIgA, and eosinophilic protein X, as well as with an increase in glutamate-producing taxa such as *Lactobacillus*, *Bifidobacterium*, and *Odoribacter* spp. [97]. These findings suggest that changes in the microbiota composition may influence disease progression rather than disease onset. Animal studies have further supported early dysbiosis and metabolic shifts. A notable decrease in *Akkermansia muciniphila*, a species involved in gut barrier maintenance, has been observed in ALS models, whereas *Ruminococcus torques* and *Parabacteroides distasonis* have been linked to symptom exacerbation [69]. Although some *Ruminococcus* species synthesize beneficial butyrate, others, such as *R. gnavus*, have been associated with GI diseases, complicating their role in ALS [97]. An emerging hypothesis is that certain gut-derived neurotoxins may act as environmental triggers of ALS. *Clostridium* species, known producers of tetanus and botulinum neurotoxins, are hypothesized to contribute to ALS under specific conditions by targeting the motor neurons [114]. Collectively, these studies highlight the complex and dynamic relationship between the gut microbiome and ALS progression. Continued investigation of microbiome composition, function, and metabolite production may yield novel diagnostic and therapeutic strategies.

## 5. Therapeutic Perspectives: Targeting the Microbiome

### Microbiota-Targeted Therapies in ALS: Potential of Prebiotics, Probiotics, Postbiotics, and Synbiotics

**Figure 5:**
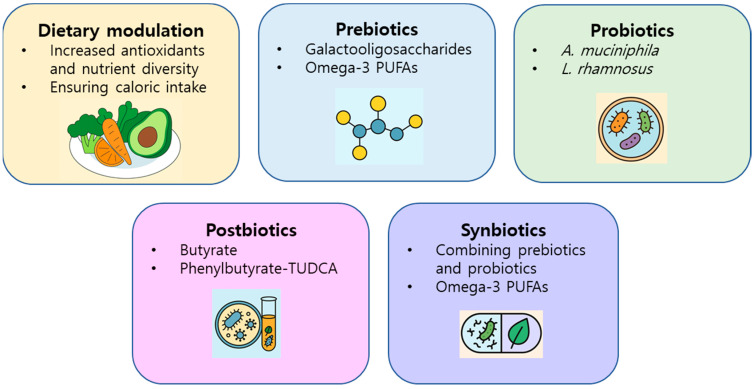
An infographic showing the potential of microbiota-targeted therapies for amyotrophic lateral sclerosis. Interventions are categorized into five groups: dietary modulation (increasing antioxidants and caloric intake), prebiotics (e.g., galactooligosaccharides and omega-3 polyunsaturated fatty acids), probiotics (*A. muciniphila* and *L*. *rhamnosus*), postbiotics (e.g., butyrate and TUDCA), and synbiotics (combinations of probiotics and prebiotics), emphasizing their roles in modulating short-chain fatty acids and supporting gut–brain axis health.

If gut-derived toxins contribute to ALS pathogenesis, identifying therapeutic interventions to prevent or mitigate their effects is critical, especially in pre-symptomatic individuals with genetic risk. Dietary modulation is a promising approach for microbiome-targeted intervention (Figure 5). Diet is a primary determinant of gut microbial composition, and increased consumption of vegetables and greater dietary diversity are recommended to maintain microbiome health [115].

The COSMOS study reported that patients with ALS with higher antioxidant and carotene intake from vegetables demonstrated better functional outcomes, including ALSFRS-R and forced vital capacity scores [116]. Another study suggested that a relatively high intake of animal-based fats and proteins may prolong survival in patients with ALS [117]. Both studies emphasized the role of a diverse and nutrient-rich diet, which is likely to enhance gut microbiota diversity [118]. Nonetheless, patients with ALS face unique nutritional challenges owing to increased metabolic demands and swallowing difficulties. Thus, any dietary recommendations must also ensure sufficient caloric intake, highlighting the need for further clinical research in this area.

Interventions targeting the microbiota have therapeutic potential (Figure 5). Prebiotics, such as galactooligosaccharides and omega-3 polyunsaturated fatty acids (PUFAs), have shown neuroprotective effects in ALS models, although the outcomes vary according to sex and compound [119,120,121,122]. Clinical studies have linked higher dietary ALA levels with slower disease progression and longer survival [119,123]. Probiotics have shown benefits in animals, but have limited effects in humans (human trials have shown only modest microbial shifts and no clear functional improvements) [124,125,126]; *A. muciniphila* has improved motor symptoms and metabolic balance in mice with ALS by increasing nicotinamide availability [124], and *Lacticaseibacillus rhamnosus* has shown neuroprotection via fatty acid oxidation pathways in *C. elegans* models. Postbiotics such as butyrate and phenylbutyrate–TUDCA have demonstrated anti-inflammatory and neuroprotective effects, with clinical trials showing modest benefits [124]; butyrate restored microbial homeostasis and reduced SOD1 aggregation in ALS models, and a phase 2 clinical trial of phenylbutyrate–TUDCA showed slowed functional decline and reduced inflammatory biomarkers in patients with ALS, supporting further investigation in phase 3 trials.

Although synbiotics remain untested in patients with ALS, findings in Alzheimer’s and Parkinson’s models are encouraging [127,128,129,130,131]. Targeting protective microbial species such as *Proteobacteria* and *Lactobacilli* may enhance therapeutic strategies [129,130]. Synbiotics combining *Lactobacillus* strains and prebiotics may enhance neuroprotection, reduce cytokine levels, and improve GBA regulation [128,129,132,133]. Moreover, microbial strains such as *Proteobacteria* may aid in the degradation of gut-derived neurotoxins and contribute to butyrate production, thereby offering an additional therapeutic approach [132,134].

Dietary modulation remains a foundational approach, with antioxidant-rich and diverse diets associated with improved clinical scores and delayed progression [116,117,118]. However, the caloric requirements in ALS complicate dietary recommendations, highlighting the need for individualized strategies and further trials. Overall, microbiota-targeted interventions, including prebiotics, probiotics, postbiotics, and synbiotics, are emerging and multifaceted strategies for ALS management. Further translational research is essential to validate these approaches and develop effective microbiome-based therapies.

## 6. Conclusions and Future Perspectives

Although previous reviews have discussed either the GBA or gut microbiota in relation to neurodegenerative diseases, this review uniquely integrates recent evidence to provide an ALS-specific mechanistic exploration of the brain–gut–microbiota axis. It synthesizes insights from neural, immune, and endocrine pathways, highlights underexplored components such as the gut virome and mycobiome, and critically evaluates both positive and negative clinical findings. Furthermore, it outlines emerging microbiota-targeted therapeutic strategies, including synbiotics, postbiotics, and SCFA-based approaches, that have not been extensively discussed in earlier literature. Collectively, this review bridges the gap between foundational microbiome research and therapeutic innovation in ALS, providing original insights and expert evaluation beyond descriptive summaries.

Emerging evidence suggests that gut-derived neurotoxins, such as FA, D-serine, and SAA metabolites, may contribute to ALS pathogenesis. These molecules are neurotoxic and elevated in some patients with ALS and can be produced by the gut microbiota [133,135,136,137,138]. Their involvement may represent a component of the multifactorial process of neurodegeneration. However, they are not exclusive to ALS and have been implicated in other neurodegenerative diseases [139,140,141,142,143].

Importantly, although microbial toxin production may exacerbate ALS, gut dysbiosis may also influence ALS through immune modulation, gut barrier dysfunction, and neurotransmitter imbalance [144]. Because environmental and non-genetic factors are potentially modifiable, understanding microbial contributions offers a valuable therapeutic approach. Preliminary findings on microbiome-targeted therapies, including prebiotics, probiotics, postbiotics, and high-dose B12, are promising; however, further research is required. Well-designed, large-scale studies that integrate gut microbiota profiling, toxin quantification, and dietary assessment are crucial for determining causal links and therapeutic targets in ALS.

## Data Availability

All the data are available within the article.

## Abbreviations

The following abbreviations are used in this manuscript:

ALS	Amyotrophic lateral sclerosis
GBA	Gut–brain axis
CNS	Central nervous system
ROS	Reactive oxygen species
GI	Gastrointestinal
SCFAs	Short-chain fatty acids
F/B	Firmicutes/Bacteroidetes
T2D	Type 2 diabetes
PNS	Peripheral nervous system
HDACs	Histone deacetylases
EECs	Enteroendocrine cells
GPCRs	G-protein-coupled receptors
CCK	Cholecystokinin
PYY	Peptide YY
FFAR2	Free fatty acid receptor 2
IBD	Inflammatory bowel disease
Treg	Regulatory T cell
GLP-1	Glucagon-like peptide-1
GIP	Glucose-dependent insulinotropic polypeptide
ECs	Enterochromaffin cells
5-HT	Serotonin
NTS	Nucleus tractus solitarius
GABA	Gamma-aminobutyric acid
ENS	Enteric nervous system
FMT	Fecal microbiota transplantation
GFAP	Glial fibrillary acidic protein
α-SMA	α-smooth muscle actin
ANS	Autonomic nervous system
LPSs	Lipopolysaccharides
TMAO	Trimethylamine-N-oxide
sIgA	Secretory IgA
PUFAs	Polyunsaturated fatty acids
